# Curcumin Differs from Tetrahydrocurcumin for Molecular Targets, Signaling Pathways and Cellular Responses

**DOI:** 10.3390/molecules20010185

**Published:** 2014-12-24

**Authors:** Bharat B. Aggarwal, Lokesh Deb, Sahdeo Prasad

**Affiliations:** Cytokine Research Laboratory, Department of Experimental Therapeutics, The University of Texas MD Anderson Cancer Center, Houston 77054, TX, USA; E-Mails: lokeshdeb@gmail.com (L.D.); spbiotech@gmail.com (S.P.)

**Keywords:** curcumin, tetreahydrocurcumin, antioxidant, anti-inflammatory

## Abstract

Curcumin (diferuloylmethane), a golden pigment from turmeric, has been linked with antioxidant, anti-inflammatory, anticancer, antiviral, antibacterial, and antidiabetic properties. Most of the these activities have been assigned to methoxy, hydroxyl, α,β-unsaturated carbonyl moiety or to diketone groups present in curcumin. One of the major metabolites of curcumin is tetrahydrocurcumin (THC), which lacks α,β-unsaturated carbonyl moiety and is white in color. Whether THC is superior to curcumin on a molecular level is unclear and thus is the focus of this review. Various studies suggest that curcumin is a more potent antioxidant than THC; curcumin (but not THC) can bind and inhibit numerous targets including DNA (cytosine-5)-methyltransferase-1, heme oxygenase-1, Nrf2, β-catenin, cyclooxygenase-2, NF-kappaB, inducible nitric oxide synthase, nitric oxide, amyloid plaques, reactive oxygen species, vascular endothelial growth factor, cyclin D1, glutathione, P300/CBP, 5-lipoxygenase, cytosolic phospholipase A2, prostaglandin E2, inhibitor of NF-kappaB kinase-1, -2, P38MAPK, p-Tau, tumor necrosis factor-α, forkhead box O3a, CRAC; curcumin can inhibit tumor cell growth and suppress cellular entry of viruses such as influenza A virus and hepatitis C virus much more effectively than THC; curcumin affects membrane mobility; and curcumin is also more effective than THC in suppressing phorbol-ester-induced tumor promotion. Other studies, however, suggest that THC is superior to curcumin for induction of GSH peroxidase, glutathione-S-transferase, NADPH: quinone reductase, and quenching of free radicals. Most studies have indicated that THC exhibits higher antioxidant activity, but curcumin exhibits both pro-oxidant and antioxidant properties.

## 1. Introduction

Curcumin, or diferuloylmethane, is a yellow crystalline substance that is isolated from turmeric (*Curcuma longa*). It is known to exhibit pleiotropic activities that include antioxidant, anti-inflammatory, antiviral, antifungal, antibacterial, anticancer, antidiabetic, and neuroprotective properties [1,2,3]. Two other curcuminoids are desmethoxycurcumin and bis-desmethoxycurcumin. Curcuminoids are natural phenols that are responsible for the yellow color of turmeric [4,5,6]. Curcumin can be used for boron quantification in the curcumin method. It reacts with boric acid to form a red compound, rosocyanine [7]. Curcumin can exist in at least two tautomeric forms, keto and enol. The enol form is more energetically stable in the solid phase and in solution [8]. Curcumin incorporates several functional groups. The aromatic ring systems, which are polyphenols, are connected by two α,β-unsaturated carbonyl groups. The diketones form stable enols or, because they are easily deprotonated, form enolates, whereas the α,β-unsaturated carbonyl is a good Michael acceptor and undergoes nucleophilic addition [9]. The structure was first identified in 1910 by J. Miłobędzka, Stanisław Kostanecki, and Wiktor Lampe.

**Figure 1:**
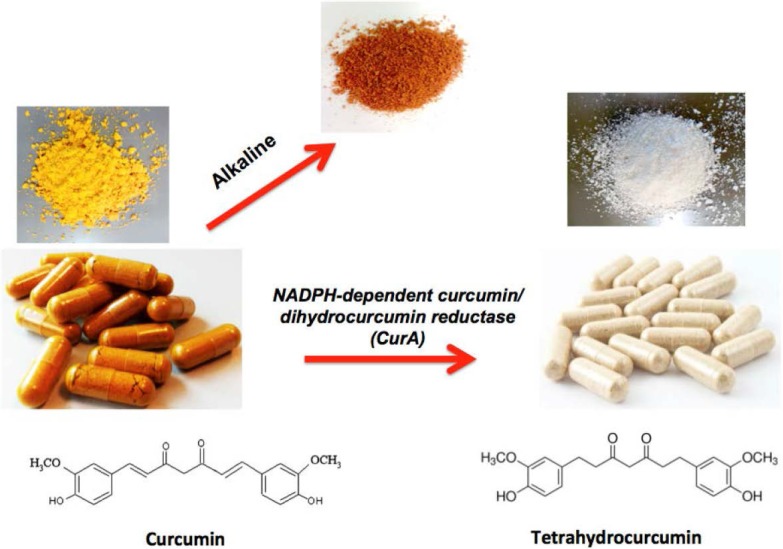
With increasing pH (alkalinity), curcumin changes to red. Curcumin metabolically converts to tetrahydrocurcumin by using the NADPH-dependent curcumin/dihydrocurcumin reductase (CurA) enzyme. Structurally, curcumin has the α,β-unsaturated carbonyl group, but tetrahydrocurcumin lacks α,β dienes.

Tetrahydrocurcumin (THC), a colorless or white metabolite of curcumin (Figure 1), was first detected in 1978 by Holder *et al*. [10]. THC has been shown to exhibit pharmacological activities similar to those of curcumin. Besides THC, other metabolites of curcumin have been identified, including the conjugates curcumin glucuronide and curcumin sulfate, which have been shown to be biologically inactive. In contrast, THC has been shown to be quite active in mediating activities similar to those of curcumin. Curcumin-converting microorganisms have been identified in human feces, with *Escherichia coli* exhibiting the highest activity [11]. The curcumin-converting enzyme purified from *E. coli*, with a molecular mass of about 82 kDa and consisting of two identical subunits, preferentially acts on curcumin through nicotinamide adenine dinucleotide phosphate (reduced form) (NADPH), converting it to THC. Whether curcumin is more active or less active than THC is not known and is thus the focus of this review.

Curcumin contains the α,β-unsaturated carbonyl group, but THC, which lacks α,β dienes, is unable to form Michael adducts with intracellular proteins (Figure 1). Curcumin may disrupt disulfide bond formation by the electrophilic dienone. Free thiols on cysteine-rich proteins are available to react with Michael acceptors of curcumin but not with THC.

Studies have shown that curcumin given intraperitoneally is first biotransformed to dihydrocurcumin and then to THC [12]. THC, one of the major metabolites of curcumin, was very stable in 0.1 M phosphate buffers at various pH values and was more stable than curcumin in 0.1 M phosphate buffer at pH 7.2 (37 °C). In a study of the metabolism of curcumin in human and rat intestine, curcumin underwent extensive reduction in the gastrointestinal tract; furthermore, curcumin’s metabolic conversion to THC was more extensive in human than in rat intestinal tissue [13].

Besides THC, various other metabolites of curcumin are dihydrocurcumin (DHC), hexahydrocurcumin (HHC), octahydrocurcumin (OHC), curcumin glucuronide, DHC-glucuronide, THC-glucuronide, and curcumin sulfate. Some of these metabolites are also reported to have anti-inflammatory and antioxidant properties [1,14]. In a study, HHC has shown to exhibit anti-inflammatory activity by inhibiting COX-2 expression, which was comparatively lesser than curcumin [15] and antioxidant activity in murine macrophages [16]. HHC has shown to induce cytotoxicity by a massive accumulation of SW480 cells in the G1/G0 phase of the cell cycle [17] and synergistically inhibit the growth of HT-29 colorectal cancer cells with 5-fluorouracil [18].

OHC also has anti-inflammatory and antioxidant properties. It suppressed NF-κB activity much lesser than curcumin [19]. However, it exhibits antioxidant activity by suppressing the AAPH-induced linoleic oxidation and DPPH scavenging activity higher than curcumin [20]. Another metabolite curcumin sulfate has shown biological activity but lesser than curcumin, specifically, in the inhibition of PGE2 activity [15].

## 2. Studies Showing Curcumin to Be More Active than THC

Curcumin and THC have both been shown to have several biological activities. However, numerous *in vitro* and animal studies have shown that curcumin is more active than THC (Table 1). These comparative biological activities include antioxidant, anti-inflammatory, anticancer, antiviral, neurological, and immunological properties.

**Table 1:** Studies showing curcumin to be more active than THC.

*•Curcumin was more active than THC in suppressing carrageenin-induced inflammation [21].*
*•Curcumin was more effective than THC in preventing PMA-induced skin tumor promotion in mice [22].*
*•Curcumin was more effective than THC as an antioxidant [23].*
*•Under aerated conditions, curcumin was more active than THC [24].*
*•Curcumin was more effective than THC in suppressing NF-κB activation [19,25,26].*
*•Curcumin was more effective than THC in down-modulating PMA-induced COX2 expression and PGE2 production [15].*
*•Curcumin was more effective than THC in inhibiting 5-LOX activity [27].*
*•Curcumin was more active than THC in ROS production and as a membrane mobility coefficient [28].*
*•Curcumin was more effective than THC in modulating ABC drug transporters [29].*
*•Curcumin induced apoptosis of HL-60 (decrease of bcl-2; increase of bax) but THC did not [30].*
*•Curcumin induced HO-1 expression through activation of ARE but THC did not [31].*
*During induction of cell death, curcumin induced ROS and GSH; THC did not [32]*
*•Curcumin, but not THC, inhibited NO production and iNOS expression [26].*
*•Curcumin was more effective than THC in inhibiting the Wnt/beta-catenin pathway by decreasing the amount of the transcriptional coactivator p300 [33,34].*
*•Curcumin, but not THC, inhibited LPS-stimulated NF-κB and COX-2 gene expression [35].*
*•Curcumin, but not THC, was effective in reducing amyloid plaque burden and amyloid aggregation [36].*
*•Curcumin, but not THC, induced HO-1 expression and Nrf2 nuclear translocation [37]*
*•Curcumin, but not THC, covalently blocked the catalytic thiolate of C1226 of DNMT1 [38].*
*•Curcumin, but not THC, inhibited Ca(2^+^) influx through CRAC for activating immune cells [39].*
*•Curcumin was more effective than THC in inducing FOXO3a-mediated gene expression by inducing FOXO3a phosphorylation and nuclear translocation [40].*
*•Curcumin was more effective than THC in reducing β-amyloid and phosphorylated Tau protein burden in Alzheimer transgenic mice [41].*
*•Curcumin was more active than THC in suppressing LPS-induced production of TNF-α [42].*
*•Curcumin, but not THC, inhibited entry of hepatitis C virus genotypes into human liver cells [43].*
*•Curcumin, but not THC, was taken up and increased lipid accumulation in monocytic cell line THP-1 [44].*
*•Curcumin was more effective than THC in inhibiting TNF-induced expression of cyclin D1 and VEGF [25].*
*•Curcumin inhibited type A influenza virus infection to a greater extent than THC by interfering with viral hemagglutination activity [45].*
*•Curcumin inhibited IKK1 and IKK2 activities induced by LPS to a greater extent than THC [19].*

PMA, phorbol 12-myristate 13-acetate; NF-κB, nuclear factor-kappaB; COX2, cyclooxygenase-2; PGE2, prostaglandin E2; 5-LOX, 5-lipoxygenase; HAT, histone acetyltransferase; THC, tetrahydrocurcumin; HO-1, heme oxygenase 1; ARE, antioxidant response element; ROS, reactive oxygen species; GSH, glutathione; iNOS, inducible nitric oxide synthase; Nrf2, nuclear factor erythroid 2 [NF-E2]-related factor 2; LPS, lipopolysaccharides; DNMT1, DNA (cytosine-5)-methyltransferase 1; CRAC, Ca(2+)-release activated Ca(2+) channels; FOXO3a, Forkhead box O3a; TNF-α, tumor necrosis factor alpha; VEGF, vascular endothelial growth factor; IKK, IkappaB kinase.

### 2.1. Antioxidant Activities

Khopde *et al.* [24] examined curcumin and THC for gamma radiation–induced lipid peroxidation and reported that curcumin was more potent inhibitor than THC. However, when they examined curcumin and THC for N_2_O-triggered HO-induced lipid peroxidation, they found that THC was more potent than curcumin. These differences were attributed to the fact that curcumin is more lipid-soluble than THC, whereas THC is more water-soluble than curcumin. However, when the antiallergic activity of curcumin by histamine release from rat basophilic leukemia cells was examined, it was found to be comparable to that of THC [46]. This effect was found to be unrelated to antioxidant activity. When Atsumi *et al.* [32] examined curcumin and THC for cytotoxicity and ROS generation after visible light irradiation, they found that curcumin significantly reduced the intracellular glutathione (GSH) level, whereas THC had no effect (Table 1).

### 2.2. Pro-Oxidant Activities

Of interest, besides antioxidant activity, curcumin also exhibits pro-oxidant activities. Atsumi *et al.* [28] examined the relationship between intracellular ROS production and membrane mobility by using curcumin and THC in human gingival fibroblasts and human submandibular gland carcinoma cells. Curcumin produced ROS dose-dependently, which led to decreased membrane mobility. This affect was reversed by the addition of GSH. In contrast, THC had no effect on ROS production or on membrane mobility. Thus, the authors concluded that the reduction in membrane mobility induced by curcumin was attributed to ROS production. The oxidative effects of curcumin were linked to the structure of the α,β-unsaturated carbonyl moiety as well as to the phenolic OH group of this compound, since THC had no effect. Results from our laboratory also confirmed that curcumin, but not THC, can exhibit pro-oxidant activity, as indicated by the generation of ROS [25].

### 2.3. Anti-Inflammatory Activities

Mukhopadhyay *et al.* [21] were the first to compare the anti-inflammatory activity of curcumin with that of THC by using the carrageenan-induced rat paw edema assay and the cotton pellet granuloma formation test. In these models of inflammation, curcumin was quite effective in suppressing inflammation, but THC was less effective. THC completely lacked activity in the cotton pellet granuloma formation test. Of note, both curcumin and THC decreased carrageenin-induced paw edema at low doses; at higher doses of both, however, this effect was only partially reversed.

Curcumin has also been shown to affect inflammatory pathways through the modulation of lipid accumulation in monocytes/macrophages [40]. Curcumin increased the expression of two lipid transport genes, the fatty acids transporter CD36/FAT and the fatty acids binding protein 4 (FABP4/aP2), leading to increased lipid levels in cells. When the activity of Forkhead box O3a (FOXO3a), a transcription factor centrally involved in regulating several stress resistance and lipid transport genes, was examined, curcumin was shown to have doubled FOXO3a-mediated gene expression, possibly as a result of influencing FOXO3a phosphorylation and nuclear translocation. THC, in contrast, did not up-regulate CD36 or increase FOXO3a activity. Thus, the up-regulation of FOXO3a activity by curcumin, but not by THC, could be a mechanism to protect against oxidant- and lipid-induced damage in the inflammatory cells of the vascular system.

Nakagawa *et al*. [44] suggested that differential cellular uptake of curcumin and THC may be linked to the previously observed differences in their effects on lipid accumulation in macrophages. Indeed, the authors found that curcumin was readily taken up by the cells and slowly metabolized to hexahydrocurcumin sulfate but that uptake of THC was low; this finding correlated with increased lipid uptake of cells with curcumin but decreased lipid uptake of cells with THC. Thus, it is possible that curcumin and THC are taken up and metabolized differently in the cells, which determines their biological activity.

Pan *et al.* [19] showed that curcumin down-regulated the lipopolysaccharide (LPS)-induced expression of iNOS in macrophages through the down-regulation of NF-κB, whereas THC was less active. Our laboratory has shown that curcumin, but not THC, can suppress tumor necrosis factor (TNF)-α-induced NF-kB activation [25]. These results are in agreement with those reported by Murakami *et al.* [35], who compared the anti-inflammatory activities of curcumin and THC. They examined the cyclooxygenase-2 (COX-2) expression in LPS- or porphyromonas gingivalis fimbria–stimulated RAW 264.7 cells. The fimbria-stimulated expression of the COX-2 gene was inhibited by curcumin but not by THC. Similarly, LPS-stimulated COX-2 gene expression was completely inhibited by curcumin, but incompletely inhibited by THC. Curcumin blocked NF-kB activation in the cells, but THC did not. Nishida *et al.* [42] also showed that curcumin was more effective than THC in suppressing LPS-induced TNF-α production in macrophages. These results correlated with suppression of LPS-induced NF-kB activation and IkB phosphorylation by curcumin but not by THC. When examined for TNF-α-induced ROS production, THC was as active as or more effective than curcumin in suppressing ROS production.

The induction of heme oxygenase-1 (HO-1) expression has been shown to counteract various stressful events; thus pharmacologic agents that target this action have therapeutic potential. HO-1 has been shown to mediate the anti-inflammatory effects of curcumin [47]. Curcumin was found to induce HO-1 expression via activation of the nuclear factor-erythroid-2-related factor 2 (Nrf2) by binding with cysteine residue of Keap1 [48], whereas THC had no effect on HO-1 expression, on Nrf2 activation in rat vascular smooth muscle cells (VSMCs) [31], or on macrophages [37]. Curcumin was found to exhibit growth inhibitory effects on VSMC, and these effects were mediated by the up-regulation of p21 and HO-1 expression. Similarly, another study showed that curcumin, but not THC, attenuated dimethylnitrosamine-induced liver injury in rats through Nrf2-mediated induction of HO-1 [48].

Ireson *et al*. [15] reported that curcumin was quite effective in suppressing phorbol ester-induced PGE2 production in human colonic epithelial cells, whereas THC had only weak PGE2 inhibitory activity. COX2 expression plays an important role in carcinogenesis. Hong *et al*. [27] examined the effects of curcumin and THC on the release of arachidonic acid and its metabolites in the murine macrophage and in HT-29 human colon cancer cells and found that curcumin inhibited the formation of PGE2 in LPS-stimulated RAW cells and inhibited LPS-induced COX-2 expression. Curcumin and THC also potently inhibited the activity of human recombinant 5-LOX, with 50% inhibitory concentration (IC50) values of 0.7 and 3 µM, respectively. Murakami *et al*. [35] showed that LPS-stimulated expression of the COX-2 gene was inhibited by curcumin but not by THC and that THC’s lack of this effect was linked to the inability of THC to suppress LPS-induced NF-kB activation, to the chemical hardness of the two molecules, and to higher pro-oxidative activity of curcumin.

Excess production of nitric oxide (NO) by inducible NO synthase (iNOS) in activated macrophages has been linked to acute and chronic inflammation. Curcumin has been shown to inhibit NO production and iNOS expression in activated macrophages. Pae *et al*. [26] found that curcumin, but not THC, inhibited NO production, iNOS expression, and NF-kB activation; these researchers concluded that the conjugated double bonds in curcumin play an important role in its anti-inflammatory activity.

When curcumin was compared with THC for antiproliferative effects against HepG2 cells, curcumin was found to be more active (IC50 of 85.98 *vs*. 233.12 µM) [49]. When compared for antiangiogenic activity, however, THC was found to be more active than curcumin, possibly due to the higher antioxidant activity associated with THC compared with that of curcumin.

### 2.4. Anticancer Agent

Numerous studies have compared the anticancer potential of curcumin with that of THC. The Huang *et al.* [22] study was one of the earliest to show that THC is less potent than curcumin in phorbol ester–induced tumor promotion. The authors reported that THC was also less potent than curcumin was in TPA-induced inflammation of mouse ear. In bioassays associated with tumor promotion, *i.e.*, inhibition of tumor promoter–induced inflammation in mouse skin and Epstein-Barr virus activation, Nakamura *et al*. [23] showed that THC tends to show weaker inhibitory activities than curcumin does. These researchers, when examining for TPA-induced O_2_ generation in differentiated HL-60 cells, also found that the inhibitory activity of THC was weaker than that of curcumin.

When human submandibular adenocarcinoma and human gingival fibroblasts cells were examined, curcumin, but not THC, produced ROS and reduced membrane mobility [28]. This reduction in membrane mobility was reversed by GSH, indicating the critical role of ROS. With use of human breast cancer cells, Kang *et al*. [50] showed that both curcumin and THC inhibited the growth of human breast cancer cells but that curcumin was almost twice as effective as THC.

In another study, curcumin was again shown to exhibit more effective anticancer agent than THC did: curcumin induced apoptosis in human leukemia HL-60 cells, whereas THC had no effect [30]. This correlated with down-modulation of bcl-2, up-regulation of bax, release of cytochrome C, and activation of caspase-8, caspase-9, and caspase-3 by curcumin but not by THC. Increase in endoplasmic reticulum (ER) stress, as indicated by phosphorylation of PERK and its substrate eIF2a, occurred in response to curcumin but not to THC. This led to expression of GRP78/Bip and CHOP/GADD135 and cleavage of procaspase-4 by curcumin, but not by THC.

Pae *et al*. [30] also reported that in HL-60 cells, curcumin induced apoptosis and ER stress, evidenced by the survival molecules such as phosphorylated protein kinase-like ER-resident kinase, phosphorylated eukaryotic initiation factor-2alpha, glucose-regulated protein-78, and the apoptotic molecules such as caspase-4 and CCAAT/enhancer-binding protein homologous protein (CHOP). Inhibition of caspase-4 activity by z-LEVD-FMK, blockage of CHOP expression, and treatment with salubrinal, an ER inhibitor, reduced curcumin-induced apoptosis. THC, however, was found to lack all of these activities.

Actinic keratosis is the most common precancerous lesion that involves keratinocyte proliferation. Curcumin was found to suppress the growth of human keratinocytes by suppression of p44/p42 MAPK activation, enhanced activation of p38 MAPK, and p53 phosphorylation; these effects were reversed by N-acetylcysteine [51]. THC, in comparison, was much less active than native curcumin in suppressing human keratinocyte growth.

β-catenin response transcription (CRT) is known to be aberrantly activated in colorectal cancer. Curcumin has been shown to suppress CRT activated by Wnt3a without altering the level of intracellular β-catenin and to inhibit the growth of various colon cancer cells. In addition, curcumin has been shown to down-regulate p300, which is a positive regulator of the Wnt/beta-catenin pathway. THC has also been found to inhibit CRT and cell proliferation but to a much lesser extent than curcumin does [33], indicating that the conjugated bonds in the central seven-carbon chain of curcumin are essential for inhibition of the Wnt/beta-catenin pathway and for the antiproliferative activity of curcumin. As expected, THC did not affect the level of intracellular b-catenin, TCF-4, or p300, consistent with results from CRT.

Histone acetyltransferases (HATs), p300/CBP in particular, have been implicated in the growth and survival of cancer cells. Marcu *et al.* [34] found that curcumin, but not THC, is a selective HAT inhibitor. Curcumin was found to induce proteasome-dependent degradation of p300 and the closely related CBP protein. Curcumin also inhibited the acetyltransferase activity of p300. These researchers further found that only radiolabeled curcumin, but not THC, formed a covalent association with p300.

Development of multidrug resistance (MDR) in human cancers is a major problem. Modulators of MDR are being investigated to overcome resistance. Both curcumin and THC have been shown to inhibit the function of P-gp linked to MDR and significantly increase the sensitivity of cancer cells to vinblastine, mitoxantrone, and etoposide [29]. THC, however, was found to be much less effective than curcumin.

DNA methylation of cytosine residues is an epigenetic mechanism that controls gene transcription, genome stability, and genetic imprinting. This process is regulated by DNA methyltransferases (DNMT1, DNMT3a, and DNMT3b). Curcumin was found to inhibit DNMT1 [38]. Molecular docking of the interaction of curcumin with DNMT1 suggested that curcumin covalently blocks the catalytic thiolate of C1226 of DNMT1 to exert its inhibitory effect. In addition, curcumin was found to induce global DNA hypomethylation in leukemic cells. The binding affinity of curcumin and THC, however, was found to be comparable.

### 2.5. Antiviral Activity

Hepatitis C virus (HCV) infection causes severe liver disease and affects more than 160 million individuals worldwide. People undergoing liver organ transplantation face universal reinfection of the graft. Therefore, affordable antiviral strategies targeting the early stages of infection are urgently needed to prevent the recurrence of HCV infection. Although curcumin did not affect HCV replication, it inhibited HCV entry independent of the genotype and in primary human hepatocytes by affecting membrane fluidity, thereby impairing virus binding and fusion [43]. THC under these conditions was found to have no activity. Curcumin has been shown to insert deep into the membrane in a transbilayer orientation, anchored by hydrogen bonding to the phosphate group of lipids in a manner analogous to cholesterol [52]. Studies such as that of Lupberger *et al*. [53] have indicated that EGFR as a host factor is required for HCV entry and that erlotinib, an EGFR inhibitor, suppresses HCV entry. Because curcumin has been shown to down-regulate EGFR, this may also contribute to HCV entry.

Curcumin has been reported to inhibit type A influenza virus (IAV) infection by interfering with viral hemagglutination (HA) activity [54,55], whereas THC has been less effective for suppression of IAV infection [45]. Further studies indicated that curcumin, but not THC, harbors the HA inhibitory effect. Moreover, simulation docking of curcumin with the HA structure revealed that curcumin binds to the region constituting sialic acid anchoring residues, supporting results obtained by the inhibition of HA activity. These studies indicate that the presence of the double bonds in the central seven-carbon chain enhanced the curcumin-dependent anti-IAV activity and interfered with IAV entry by curcumin’s interaction with the receptor binding region of viral HA protein.

### 2.6. Neurologic Effects

Curcumin has been shown to reduce the beta amyloid and phosphorylated tau protein burden in Alzheimer transgenic mice [41]. THC, however, was less effective in reducing phosphorylated tau protein and failed to significantly change the plaque burden or cytokine expression. Curcumin has been shown to both suppress inflammatory response and promote the shift from Th1 to Th2 immunity [56] as indicated by the expression of IL-2 and IL-4. Again THC was much less effective than curcumin in inducing the expression of these cytokines.

Begum *et al.* [36] compared the antioxidant, anti-inflammatory, and anti-amyloidogenic effects of curcumin with those of THC by administering each agent chronically to aged Tg2576 APPsw mice or acutely to LPS-injected wild-type mice. Higher drug plasma levels were noted after THC compared with curcumin gavage; resulting brain levels of parent compounds were similar, correlating with reduction in LPS-stimulated iNOS, nitrotyrosine, F2 isoprostanes, and carbonyls. In both acute (LPS) and chronic (Tg2576) inflammation, THC and curcumin similarly reduced IL-1beta. Despite these similarities, only curcumin was effective in reducing amyloid plaque burden, insoluble beta-amyloid peptide (Abeta), and carbonyls. THC had no effect on plaques or insoluble Abeta, but reduced both Tris-buffered saline-soluble Abeta and phospho-JNK. Curcumin, but not THC, prevented Abeta aggregation. The THC metabolite, however, was detected in brain and plasma from mice that had been chronically fed the parent compound. These data suggest that the dienone bridge that is present in curcumin, but not in THC, is necessary to reduce plaque deposition and protein oxidation in an Alzheimer model.

Rajeswari and Sabesan [57] compared the effects of curcumin with those of THC in a model of Parkinson disease induced in mice by 1-methyl-4-phenyl-1,2,3,6-tetrahydropyridine (MPTP). In this model, depletion of dopamine (DA) and DOPAC (3,4-dihydroxy phenyl acetic acid) occurred with increased monoamine oxidase (MAO-B) activity. Curcumin and THC, systemically administered, were equally effective in reversing the MPTP-induced depletion of DA and DOPAC and thus were equally effective for neuroprotection against MPTP-induced neurotoxicity.

### 2.7. Immunological Effects

Ca^2+^ influx through Ca^2+^-release activated Ca^2+^ channels (CRAC) is critical for activating immune cells. Curcumin was shown to be very effective for inhibition of CRAC, and this inhibition was mediated through the cysteine residue at position 195 in CARC, since replacement of this residue with serine reversed the effects [39]. Of interest, THC was found to be very weak for inhibition of CRAC.

## 3. Studies Showing THC to Be More Active than Curcumin

Several other comparative studies on curcumin and THC revealed that THC is as active as curcumin and in some cases is more active than curcumin (Table 2). These findings were supported by various studies.

**Table 2:** Studies showing THC to be more active than curcumin.

*•THC was more active than curcumin in the carrageenin-induced rat paw edema test for anti-inflammatory activity [21]*
*•THC was more active than curcumin as an antioxidant [20,58,59,60,61]*
*•THC was more active than curcumin for suppression of lipid peroxidation of erythrocyte membrane ghosts [62].*
*•THC was more active than curcumin for prevention of DMH-induced ACF formation in mice [63].*
*•THC was more active than curcumin for suppression of radiation-induced lipid peroxidation [24].*
*•THC was more active than curcumin for suppression of nitrilotriacetate-induced oxidative renal damage [64].*
*•THC was more active than curcumin for suppression of LDL oxidation [65].*
*•THC was more active than curcumin for inhibition of COX2-dependent arachidonic acid metabolism [27].*
*•THC was equal to curcumin in potency for suppression of histamine release [46].*
*•THC was more active than curcumin for inhibition of JNK activation [36].*
*•THC was more active than curcumin for protection from chloroquine-induced hepatotoxicity in rats [66].*
*•THC was more active than curcumin in normalizing blood glucose and improvement of altered carbohydrate metabolic enzymes in diabetic animals [67].*
*•THC was more active than curcumin for antidiabetic effects in rats [59].*
*•THC was more active than curcumin in increasing plasma insulin in diabetic rats [59,67,68]*
*•THC was more active than curcumin in preventing brain lipid peroxidation in diabetic rats [69].*
*•THC was more active than curcumin in increasing tissue sialic acid [67].*
*•THC was more active than curcumin for antidiabetic and antihyperlipidemic effects [70]*
*•THC was more active than curcumin in reducing accumulation and cross-linking of collagen in diabetic rats [71].*
*THC was more active than curcumin in modulating renal and hepatic functional markers in diabetic rats [72]*
*•THC was more active than curcumin in modulating erythrocyte TBARS in diabetic rats [59].*
*•THC was more active than curcumin in a hepatoprotective role in CCL4-induced liver damage in rats and alcoholic liver disease model rats [73].*
*•THC was more effective than curcumin in improving the specific insulin binding to the receptors on erythrocytes [68].*
*•THC was more active than curcumin in binding to phospholipase (PLA) 2 [74]*
*•THC was more active than curcumin in preventing azoxymethane-induced colon carcinogenesis [75].*
*•THC was more active than curcumin as an antihypertensive [61].*
*•THC activated p53 and p21 more effectively than curcumin [51].*

DMH, 1, 2-dimethylhydrazine; ACF, aberrant crypt foci; LDL, low-density lipoprotein; COX2, cyclooxygenage-2; THC, tetrahydrocurcumin; JNK, c-Jun N-terminal kinases; TBARS, thiobarbituric acid reactive substances; CCl4, carbon tetrachloride

### 3.1. Antioxidant Activities

Naito *et al.* [65], who examined copper-induced oxidation of human low-density lipoprotein (LDL) *in vitro*, found that THC was as potent as α-tocopherol but more potent than curcumin. Sugiyama *et al.* [62], in studying antioxidant activity by lipid peroxidation of erythrocyte membrane ghosts, also found that THC was more potent than curcumin. They further showed that β-diketone moiety of THC must exhibit antioxidative activity by cleavage of the C-C bond at the active methylene carbon between two carbonyls in the beta-diketone moiety. THC was also found to be more potent than curcumin by Somparn *et al.* [20], who used the DPPH scavenging assay to evaluate free radical scavenging activity.

### 3.2. Anti-Inflammatory Activities

In another study, Okada *et al*. [64] showed that THC ameliorates oxidative stress–induced renal injury in mice; for this activity, THC was more active than curcumin. Okada *et al*., examined the protective effects of curcumin and THC against ferric nitrilotriacetate (Fe-NTA)-induced oxidative renal damage in male ddY mice. THC significantly inhibited 2-thiobarbituric acid reactive substances (TBARS) and 4-hydroxy-2-nonenal-modified proteins and 8-hydroxy-2'-deoxyguanosine formation in the kidney; but curcumin inhibited only 4-hydroxy-2-nonenal-modified protein formation. THC was also found to be more easily absorbed from the gastrointestinal tract than curcumin. Furthermore, THC induced antioxidant enzymes, such as glutathione peroxidase, glutathione-S-transferase, and NADPH:quinone reductase, better than curcumin and scavenged Fe-NTA–induced free radicals *in vitro*.

Another study, which examined the radical-scavenging activity of curcumin and THC with thiols [60] such as 2-mercapto-1-methylimidazole, found that THC oxidized by peroxy radicals may be more antioxidative than curcumin in the interplay with GSH.

The enzyme phospholipase A2 releases arachidonic acid, which serves as a substrate for proinflammatory mediators, such as prostaglandins leucotriens. The binding of the substrate to PLA2 occurs through a well-formed hydrophobic channel. Thus, blocking the hydrophobic channel is an effective way to inhibit PLA2. Compounds inhibiting PLA2 have been implicated as potential therapeutic agents in the treatment of inflammation-related diseases. Dileep *et al*. [74] used molecular modeling and docking to compare the binding of THC to PLA2 with the binding of curcumin to PLA2 and found that THC exhibits better binding energy than curcumin. The effects of curcumin and THC on Nω-nitro-l-arginine methyl ester (l-NAME)-induced hypertension and oxidative stress in rats were examined by Nakmareong *et al*. [61], who found that THC was more potent than curcumin as an antihypertensive agent. The beneficial effects correlated with increased expression of eNOS, decreased plasma malondialdehyde levels, and increased plasma GSH levels.

### 3.3. Anticancer Effects

In animal studies, 1,2-dimethylhydrazine dihydrochloride with putative preneoplastic aberrant crypt foci were used in colon carcinogenesis models as end-point marker lesions. In these models, THC was found to be more active than curcumin in inhibiting aberrant crypt foci development and cell proliferation [63]; however, THC was less active than curcumin in inhibiting TPA-induced ornithine decarboxylase activity and tumor promotion in 7,12-dimethylbenz[a]anthracene-initiated mouse skin carcinogenesis [22].

Another study showed that THC is more effective than curcumin in preventing azoxymethane-induced colon carcinogenesis [75]. This study found that these effects were mediated by decreasing the levels of iNOS and COX-2 through down-regulation of ERK1/2 activation, AOM-induced Wnt-1 and β-catenin protein expression, and phosphorylation of GSK-3β in colonic tissue. Reduction in the protein level of connexin-43, an important molecule of gap junctions, was also noted.

### 3.4. Neurologic Effects

Acetylcholine is a neurotransmitter that is deactivated by acetylcholinesterase (AChE). Thus, inhibitors of AChE have potential for treatment of dementia and memory loss associated with neurological diseases such as Alzheimer disease. Curcumin has been shown to inhibit AChE. THC-induced inhibition of AChE exhibited an IC50 that was about two times better than that of curcumin [76].

### 3.5. Antidiabetic Effects

Oxidative stress has been linked to a wide variety of diseases including diabetes. Numerous studies have examined the effect of curcumin and THC on antioxidant status in rats with streptozotocin-nicotinamide-induced diabetes. Murugan and Pari [59] reported that oral administration of THC resulted in a significant reduction in blood glucose levels; a significant increase in plasma insulin levels; and a significant increase in the activities of superoxide dismutase, catalase, glutathione peroxidase, glutathione-*S*-transferase, reduced GSH, vitamin C, and vitamin E in the liver and kidneys of diabetic rats; they further reported significant decrease in TBARS and hydroperoxide formation in the liver and kidneys. The antidiabetic and antioxidant effects of THC were found to be more potent than were those of curcumin at the same dose. These studies thus suggest that THC is more active than curcumin for antioxidant and antidiabetic effects in type 2 diabetic rats.

Hyperlipidemia is an associated complication of diabetes mellitus. Murugan and Pari [72] also examined the lipid profile and lipid peroxidation in rats with streptozotocin-nicotinamide-induced diabetes after exposure to curcumin. They found that both curcumin and THC induced a significant reduction in blood glucose and a significant increase in plasma insulin in diabetic rats; curcumin and THC also caused a significant reduction in lipid peroxidation (TBARS and hydroperoxides) and lipids (cholesterol, triglycerides, free fatty acids, and phospholipids) in serum and tissues. Again THC was more effective than curcumin. In another report, Pari and Murugan [71] compared the effects of THC with curcumin on the lipid profile in rats with streptozotocin-nicotinamide-induced diabetes and found that both agents caused a significant increase in plasma insulin but a significant reduction in blood glucose, serum and liver cholesterol, triglycerides, free fatty acids, phospholipids, HMG CoA reductase activity, and very low-density lipoprotein and LDL cholesterol levels. The decreased serum high-density lipoprotein cholesterol in diabetic rats was also reversed. These results indicated that both THC and curcumin had antihyperlipidemic action in control and experimental diabetic rats but that THC was more potent than curcumin at the same dose.

In another study, the same investigators examined the effect of THC and curcumin on hepatic and renal functional markers and protein levels in experimental rats with type 2 diabetes [72]. THC and curcumin brought back to near normal the total protein, albumin, and globulin levels and the albumin/globulin ratio; they also reversed the activities of hepatic and renal markers. Again, the protective effect of THC was better than that of curcumin.

When the effects of curcumin and THC on erythrocyte membrane-bound enzymes and antioxidant activity were examined in a streptozotocin-nicotinamide-induced type 2 diabetes rat model, both curcumin and THC were shown to decrease levels of blood glucose, glycosylated hemoglobin, and erythrocyte TBARS and increase levels of plasma insulin, hemoglobin, and erythrocyte antioxidants [59]. For all of these activities, THC was again more potent than curcumin.

Collagen is an important constituent of most of the tissues that are affected in diabetic patients. Modifications of this protein may play a critical role in the complication of diabetes. In experimental diabetes, collagen content usually increases with extensive modifications in characteristics such as extent of glycation, cross-linking, and collagen-linked fluorescence. Pari and Murugan [71] examined the effects of THC and curcumin on tail tendon collagen in rats with type 2 diabetes that was induced in a streptozotocin-nicotinamide model and found that in diabetic rats, hydroxyproline, collagen content, and degree of cross-linking were increased. They also found that administration of THC or curcumin to diabetic rats for 45 days significantly reduced the accumulation and cross-linking of collagen; furthermore, THC was found to be more effective than curcumin.

Pari and Murugan also examined the effect of THC and curcumin on the occurrence of oxidative stress in the brains of rats with diabetes and found that oral administration of THC/curcumin (80 mg/kg of body weight) to diabetic rats for 45 days significantly reduced blood glucose; increased plasma insulin levels; increased the activities of superoxide dismutase, catalase, glutathione peroxidase, glutathione-*S*-transferase; and reduced GSH in the brains of diabetic rats. A decrease in the lipid peroxidative markers thiobarbituric acid-reactive substances and hydroperoxides in the brain was also noted. Again THC was more effective than curcumin [59].

With use of circulating erythrocytes, the effect of curcumin on insulin-binding has been compared with that of THC in type 2 diabetic rats [68]; THC significantly improved specific insulin binding to the receptors through a significant increase in plasma insulin, and the effect of THC was more prominent than that of curcumin.

### 3.6. Other Effects

Chloroquine (CQ) is a synthetic quinoline drug commonly used in treatment of malaria and other diseases such as extraintestinal amebiasis, gout, and rheumatoid arthritis. Treatment with CQ is accompanied by adverse effects such as gastrointestinal upset, headache, visual disturbances, cardiotoxic action, liver damage, and hepatitis. When examined for the protective effect on CQ-induced hepatotoxicity in rats, THC was found to be more effective than curcumin [77]. Decreases in levels of lipid peroxides and hydroperoxides were greater with THC than with curcumin; increases in levels of non-enzymatic antioxidants (vitamin C, vitamin E, and reduced GSH) and enzymatic antioxidants (superoxide dismutase, catalase, and glutathione peroxidase) were higher with THC than with curcumin. Curcumin was also found to be less active than THC in protecting rats from CQ-induced nephrotoxicity [77].

## 4. Bioavailability of Curcumin and THC

Curcumin has shown to undergoes metabolic *O*-conjugation to curcumin glucuronide and curcumin sulfate and bioreduction to THC, hexahydrocurcumin, and hexahydrocurcuminol in animals [12,78]. Because of this, the oral bioavailability of curcumin has found extremely low in *in vivo*. However, curcumin’s active metabolic component THC is found bioavailable in tissue and plasma. In a study, Curcuma-P^®^ administration in animals for 4 weeks showed a presence of THC (235 ± 78 ng/100 mg tissue) but not curcumin inside the subcutaneous adipose tissue. However, neither THC nor curcumin were detected in the plasma of mice [79]. THC have been detected in rats’ intestinal and hepatic cytosol. It is regarded as the available forms of curcumin *in vivo* because it is more stable than curcumin in buffer solutions of physiologic (pH 7.2) and also stable in plasma [12,80]. In plasma of mice treated with curcumin, no detectable curcumin was found, but THC appeared after 80 min. On the other hand, curcumin was detected in brain of curcumin-treated mice. This phenomenon was also observed in a published study of mice fed curcumin [36].

Besides these, THC is found to be more stable than curcumin. The degradation half lives of curcumin, and THC were 186 and 813 min respectively in cell culture medium. In plasma, their respective half-lives were 111 and 232 min [81]. It has been also reported that THC and curcumin increased Epigallocatechin-3-gallate uptake by greater than two-fold in MDCKII/MRP1 and HT-29 cells [82].

However, numerous studies conducted on bioavailability of curcumin and found that certain amount of curcumin are also bioavailable in serum of animals. For instance, when curcumin was administered orally to rats at a dose of 2 g/kg to rats, the serum concentration of 1.35 ± 0.23 µg/mL was observed at time 0.83 h, whereas in humans the same dose of curcumin resulted in either undetectable or extremely low serum levels [83]. Further in a human clinical trial, 3.6 g of curcumin via oral route was found to produce a plasma curcumin level of 11.1 nmol/L after an hour of dosing [84]. Other than oral route, intravenous administration of curcumin showed enough availability of curcumin in blood plasma. The concentration was 6.6 µg/mL of blood plasma when administered 2 mg/kg through tail vain [85].

## 5. Conclusions

**Figure 2:**
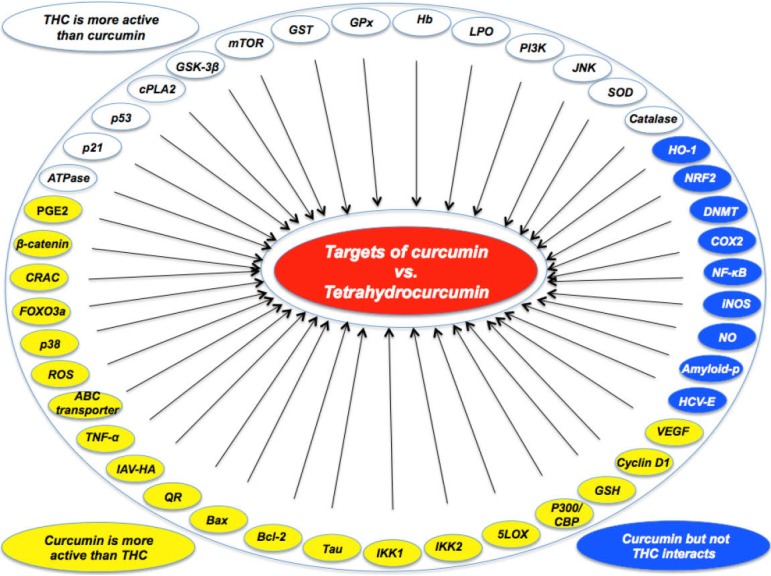
Molecular targets of curcumin vs tetrahydrocurcumin. Curcumin is more effective in modulating some targets, but tetrahydrocurcumin is more effective in others. Some molecules are modulated only by curcumin and not by tetrahydrocurcumin.

From the findings reported in this review, it is clear that curcumin, the yellow component of turmeric, is metabolized to white THC and that these two compounds exhibit distinct activities (Figure 2). Curcumin appears to bind to and modulate the activities of a wide variety of targets. THC, however, appears to be a superior antioxidant that lacks both anti-inflammatory and pro-oxidant activities. Curcumin contains a number of functional groups that are involved in target modulation. More *in vivo* data, especially clinical trials, are needed to determine which is a better molecule.

Because of the issue of bioavailability with the curcumin, introduction of THC is necessitated. THC is metabolite of curcumin and it is bioavailable after administration in the animals. However, there are limited studies are available on THC, thus more *in vitro*, *in vivo* and clinical studies are required to support the efficacy of THC against human diseases. In spite of these, designing formulations of the parent compound is also very much desired. These formulations can enhance the biological activity of curcumin and THC.
